# Supplementary material for: Functional recurrent laryngeal nerve regeneration using a silicon tube containing a collagen gel in a rat model
Source: PLoS One. 2020 Aug 27;15(8):e0237231. doi: 10.1371/journal.pone.0237231 (PMC7451556; doi:10.1371/journal.pone.0237231)
Supplement: S1 Table — (DOCX) [file pone.0237231.s001.docx]

**S1 Table. Diameter of the regenerated tissues in the tubes (µm)**

|  |  | 1mm | 3mm | 5mm |
| --- | --- | --- | --- | --- |
|  |  | 356.3 | 274.9 | 198.5 |
|  |  | 290.1 | 325.8 | 213.8 |
|  |  | 320.7 | 234.1 | 152.7 |
|  |  | 285.0 | 244.3 | 203.6 |
|  |  | 376.7 | 244.3 | 130.5 |
|  |  | 458.1 | 335.9 | 264.7 |
|  |  | 326.0 | 152.7 | 224.0 |
|  |  | 380.0 | 147.6 | 158.8 |
|  |  | 443.2 | 276.7 | 342.1 |
|  |  | 382.5 | 260.2 | 173.5 |
|  |  |  | 343.1 |  |
| mean |  | 361.9 | 258.2 | 206.2 |
| standard deviation | | 55.6 | 62.4 | 58.5 |
