# Supplementary material for: Functional recurrent laryngeal nerve regeneration using a silicon tube containing a collagen gel in a rat model
Source: PLoS One. 2020 Aug 27;15(8):e0237231. doi: 10.1371/journal.pone.0237231 (PMC7451556; doi:10.1371/journal.pone.0237231)
Supplement: S2 Table — (DOCX) [file pone.0237231.s002.docx]

**S2 Table. The ratio of the area on the treated side to the area on the untreated side (T/Uratio)**

|  | 1mm | 3mm | 5mm |
| --- | --- | --- | --- |
|  | 69.8 | 68.1 | 62.0 |
|  | 84.0 | 76.4 | 61.7 |
|  | 76.8 | 64.4 | 68.6 |
|  | 79.2 | 62.2 | 67.2 |
|  | 83.0 | 74.6 | 53.4 |
|  | 70.0 | 80.0 | 71.4 |
|  | 72.8 | 64.8 | 68.3 |
|  | 75.6 | 66.0 | 55.9 |
| mean | 76.4 | 69.6 | 63.6 |
| standard deviation | 5.1 | 6.1 | 6.0 |
